# Supplementary material for: Multi‐dataset identification of innovative feature genes and molecular mechanisms in keratoconus
Source: J Cell Mol Med. 2024 Sep 19;28(18):e70079. doi: 10.1111/jcmm.70079 (PMC11412914; doi:10.1111/jcmm.70079)
Supplement: Supplementary file 2 — Material S2. [file JCMM-28-e70079-s002.zip › 7.GSEA analysis/5.RNF217-AS1/KEGG_CELL_CYCLE.html]

Details for gene set KEGG\_CELL\_CYCLE[GSEA]

|  || Dataset | RNF217-AS1.group.cls#h\_versus\_l |
| Phenotype | group.cls#h\_versus\_l |
| Upregulated in class | l |
| GeneSet | KEGG\_CELL\_CYCLE |
| Enrichment Score (ES) | -0.43427774 |
| Normalized Enrichment Score (NES) | -1.5701591 |
| Nominal p-value | 0.09019608 |
| FDR q-value | 0.33983 |
| FWER p-Value | 0.659 |
Table: GSEA Results Summary

  

Fig 1: Enrichment plot: KEGG\_CELL\_CYCLE      
 Profile of the Running ES Score & Positions of GeneSet Members on the Rank Ordered List

  

| SYMBOL | TITLE | RANK IN GENE LIST | RANK METRIC SCORE | RUNNING ES | CORE ENRICHMENT || 1 | CDKN2B | na | 2482 | 0.317 | -0.0460 | No |
| 2 | CCNE1 | na | 7161 | 0.148 | -0.1692 | No |
| 3 | CCNA1 | na | 7846 | 0.132 | -0.1783 | No |
| 4 | STAG2 | na | 8039 | 0.127 | -0.1736 | No |
| 5 | E2F3 | na | 8526 | 0.115 | -0.1783 | No |
| 6 | CDC14A | na | 8910 | 0.107 | -0.1807 | No |
| 7 | TGFB1 | na | 9166 | 0.102 | -0.1798 | No |
| 8 | YWHAE | na | 10174 | 0.083 | -0.2022 | No |
| 9 | ESPL1 | na | 10529 | 0.077 | -0.2061 | No |
| 10 | GSK3B | na | 10962 | 0.071 | -0.2129 | No |
| 11 | ZBTB17 | na | 12052 | 0.055 | -0.2399 | No |
| 12 | SMC1B | na | 12302 | 0.051 | -0.2429 | No |
| 13 | ANAPC1 | na | 12601 | 0.047 | -0.2477 | No |
| 14 | PTTG2 | na | 12847 | 0.044 | -0.2512 | No |
| 15 | GADD45A | na | 13174 | 0.040 | -0.2574 | No |
| 16 | YWHAZ | na | 13310 | 0.038 | -0.2582 | No |
| 17 | SMC1A | na | 13405 | 0.037 | -0.2579 | No |
| 18 | YWHAQ | na | 13522 | 0.036 | -0.2583 | No |
| 19 | ORC5 | na | 13580 | 0.035 | -0.2571 | No |
| 20 | PTTG1 | na | 13701 | 0.034 | -0.2579 | No |
| 21 | CDK7 | na | 13711 | 0.034 | -0.2554 | No |
| 22 | CCNE2 | na | 14005 | 0.031 | -0.2614 | No |
| 23 | MDM2 | na | 14406 | 0.026 | -0.2709 | No |
| 24 | CREBBP | na | 14557 | 0.024 | -0.2732 | No |
| 25 | CDK2 | na | 14696 | 0.022 | -0.2754 | No |
| 26 | ATM | na | 14715 | 0.022 | -0.2741 | No |
| 27 | SMAD3 | na | 14794 | 0.021 | -0.2747 | No |
| 28 | ANAPC5 | na | 15025 | 0.019 | -0.2798 | No |
| 29 | ORC2 | na | 15628 | 0.013 | -0.2962 | No |
| 30 | STAG1 | na | 15868 | 0.010 | -0.3023 | No |
| 31 | FZR1 | na | 15874 | 0.010 | -0.3016 | No |
| 32 | SMAD2 | na | 16024 | 0.008 | -0.3052 | No |
| 33 | ATR | na | 16093 | 0.008 | -0.3065 | No |
| 34 | CDC23 | na | 16140 | 0.007 | -0.3073 | No |
| 35 | RBX1 | na | 16317 | 0.006 | -0.3119 | No |
| 36 | YWHAG | na | 16384 | 0.005 | -0.3134 | No |
| 37 | SFN | na | 16425 | 0.004 | -0.3142 | No |
| 38 | CDKN1B | na | 16572 | 0.003 | -0.3182 | No |
| 39 | SKP1 | na | 16603 | 0.003 | -0.3188 | No |
| 40 | ANAPC7 | na | 16717 | 0.001 | -0.3220 | No |
| 41 | SMAD4 | na | 16849 | 0.000 | -0.3258 | No |
| 42 | BUB3 | na | 16881 | -0.000 | -0.3266 | No |
| 43 | RBL2 | na | 18022 | -0.011 | -0.3587 | No |
| 44 | ANAPC11 | na | 18123 | -0.012 | -0.3606 | No |
| 45 | CDC20 | na | 18128 | -0.012 | -0.3598 | No |
| 46 | HDAC1 | na | 18248 | -0.013 | -0.3622 | No |
| 47 | CDC27 | na | 18332 | -0.014 | -0.3634 | No |
| 48 | RB1 | na | 18364 | -0.014 | -0.3632 | No |
| 49 | YWHAB | na | 18544 | -0.016 | -0.3671 | No |
| 50 | PKMYT1 | na | 18680 | -0.017 | -0.3696 | No |
| 51 | CCND1 | na | 18820 | -0.019 | -0.3721 | No |
| 52 | TFDP1 | na | 18838 | -0.019 | -0.3711 | No |
| 53 | ANAPC13 | na | 18874 | -0.019 | -0.3706 | No |
| 54 | TFDP2 | na | 19475 | -0.025 | -0.3859 | No |
| 55 | CCNH | na | 19631 | -0.027 | -0.3882 | No |
| 56 | CDC16 | na | 20405 | -0.034 | -0.4078 | No |
| 57 | E2F2 | na | 20470 | -0.035 | -0.4068 | No |
| 58 | CDKN2D | na | 20673 | -0.037 | -0.4097 | No |
| 59 | ANAPC4 | na | 20735 | -0.037 | -0.4084 | No |
| 60 | CDK1 | na | 20745 | -0.037 | -0.4057 | No |
| 61 | PRKDC | na | 20771 | -0.038 | -0.4034 | No |
| 62 | HDAC2 | na | 20836 | -0.038 | -0.4021 | No |
| 63 | CDKN2A | na | 21184 | -0.042 | -0.4088 | No |
| 64 | ANAPC10 | na | 21518 | -0.045 | -0.4147 | No |
| 65 | MCM7 | na | 21720 | -0.048 | -0.4167 | No |
| 66 | CUL1 | na | 21972 | -0.050 | -0.4198 | No |
| 67 | RAD21 | na | 21979 | -0.051 | -0.4159 | No |
| 68 | GADD45G | na | 22182 | -0.053 | -0.4175 | No |
| 69 | CDKN1C | na | 22196 | -0.053 | -0.4136 | No |
| 70 | WEE1 | na | 22325 | -0.054 | -0.4129 | No |
| 71 | ORC3 | na | 22634 | -0.058 | -0.4171 | No |
| 72 | ORC4 | na | 22915 | -0.061 | -0.4203 | No |
| 73 | CDK6 | na | 23374 | -0.067 | -0.4281 | No |
| 74 | EP300 | na | 23570 | -0.069 | -0.4282 | No |
| 75 | CCNB3 | na | 23783 | -0.072 | -0.4284 | Yes |
| 76 | SMC3 | na | 23865 | -0.073 | -0.4249 | Yes |
| 77 | MAD2L1 | na | 23884 | -0.074 | -0.4194 | Yes |
| 78 | CDK4 | na | 24008 | -0.075 | -0.4169 | Yes |
| 79 | CDKN1A | na | 24177 | -0.078 | -0.4154 | Yes |
| 80 | MAD2L2 | na | 24443 | -0.082 | -0.4164 | Yes |
| 81 | TGFB2 | na | 24475 | -0.082 | -0.4107 | Yes |
| 82 | CDC25B | na | 24572 | -0.084 | -0.4067 | Yes |
| 83 | CDC26 | na | 24588 | -0.084 | -0.4003 | Yes |
| 84 | RBL1 | na | 24761 | -0.087 | -0.3983 | Yes |
| 85 | PCNA | na | 24885 | -0.089 | -0.3947 | Yes |
| 86 | CCNB1 | na | 24923 | -0.089 | -0.3885 | Yes |
| 87 | YWHAH | na | 25403 | -0.097 | -0.3945 | Yes |
| 88 | MCM3 | na | 25627 | -0.100 | -0.3928 | Yes |
| 89 | CCNB2 | na | 26396 | -0.114 | -0.4057 | Yes |
| 90 | CCND3 | na | 26722 | -0.120 | -0.4054 | Yes |
| 91 | CDC7 | na | 26870 | -0.123 | -0.3997 | Yes |
| 92 | DBF4 | na | 26884 | -0.123 | -0.3901 | Yes |
| 93 | MAD1L1 | na | 27023 | -0.126 | -0.3838 | Yes |
| 94 | SKP2 | na | 27110 | -0.128 | -0.3759 | Yes |
| 95 | MCM5 | na | 27348 | -0.133 | -0.3720 | Yes |
| 96 | CHEK2 | na | 27683 | -0.141 | -0.3703 | Yes |
| 97 | ANAPC2 | na | 27696 | -0.141 | -0.3592 | Yes |
| 98 | BUB1 | na | 27755 | -0.143 | -0.3493 | Yes |
| 99 | MCM4 | na | 28135 | -0.152 | -0.3480 | Yes |
| 100 | E2F4 | na | 28238 | -0.155 | -0.3384 | Yes |
| 101 | GADD45B | na | 28272 | -0.155 | -0.3268 | Yes |
| 102 | ABL1 | na | 28463 | -0.161 | -0.3192 | Yes |
| 103 | CDC14B | na | 28615 | -0.165 | -0.3102 | Yes |
| 104 | TTK | na | 28760 | -0.170 | -0.3006 | Yes |
| 105 | WEE2 | na | 28913 | -0.174 | -0.2909 | Yes |
| 106 | CDKN2C | na | 28997 | -0.176 | -0.2791 | Yes |
| 107 | CDC45 | na | 29170 | -0.181 | -0.2694 | Yes |
| 108 | MCM6 | na | 29532 | -0.192 | -0.2643 | Yes |
| 109 | CCND2 | na | 30134 | -0.211 | -0.2645 | Yes |
| 110 | MCM2 | na | 30570 | -0.226 | -0.2588 | Yes |
| 111 | MYC | na | 30784 | -0.234 | -0.2461 | Yes |
| 112 | E2F1 | na | 31302 | -0.254 | -0.2404 | Yes |
| 113 | CCNA2 | na | 31442 | -0.260 | -0.2233 | Yes |
| 114 | ORC6 | na | 31712 | -0.274 | -0.2089 | Yes |
| 115 | E2F5 | na | 31895 | -0.284 | -0.1911 | Yes |
| 116 | ORC1 | na | 32032 | -0.293 | -0.1713 | Yes |
| 117 | BUB1B | na | 32045 | -0.294 | -0.1478 | Yes |
| 118 | TGFB3 | na | 32072 | -0.296 | -0.1246 | Yes |
| 119 | CHEK1 | na | 32222 | -0.304 | -0.1043 | Yes |
| 120 | TP53 | na | 32227 | -0.304 | -0.0797 | Yes |
| 121 | PLK1 | na | 32706 | -0.338 | -0.0662 | Yes |
| 122 | CDC25A | na | 33912 | -0.465 | -0.0633 | Yes |
| 123 | CDC6 | na | 34028 | -0.488 | -0.0271 | Yes |
| 124 | CDC25C | na | 34368 | -0.585 | 0.0104 | Yes |
Table: GSEA details [plain text format]

  

Fig 2: KEGG\_CELL\_CYCLE      
 Blue-Pink O' Gram in the Space of the Analyzed GeneSet

  

Fig 3: KEGG\_CELL\_CYCLE: Random ES distribution      
 Gene set null distribution of ES for **KEGG\_CELL\_CYCLE**
